# High optical spin-filtering in antiferromagnetic stanene nanoribbons induced by band bending and uniaxial strain

**DOI:** 10.1038/s41598-023-39593-6

**Published:** 2023-08-08

**Authors:** F. Rahimi, A. Phirouznia

**Affiliations:** 1 Department of Physics, Azarbaijan Shahid Madani University, Tabriz, 53714-161 Iran; 2 Condensed Matter Computational Research Lab, Azarbaijan Shahid Madani University, Tabriz, 53714-161 Iran

**Keywords:** Nanoscience and technology, Spintronics

## Abstract

Non-equilibrium spin-polarized transport properties of antiferromagnetic stanene nanoribbons are theoretically studied under the combining effect of a normal electric field and linearly polarized irradiation based on the tight-binding model at room temperature. Due to the existence of spin-orbit coupling in stanene lattice, applying normal electric field leads to splitting of band degeneracy of spin-resolved energy levels in conduction and valence bands. Furthermore, unequivalent absorption of the polarized photons at two valleys which is attributed to an antiferromagnetic exchange field results in unequal spin-polarized photocurrent for spin-up and spin-down components. Interestingly, in the presence of band bending which has been induced by edge potentials, an allowable quantum efficiency occurs over a wider wavelength region of the incident light. It is especially important that the variation of an exchange magnetic field generates spin semi-conducting behavior in the bended band structure. Moreover, it is shown that optical spin-filtering effect is obtained under the simultaneous effect of uniaxial strain and narrow edge potential.

## Introduction

Recently, spin-functionalized optoelectronics properties of nanostructures have attracted significant interest in the scientific community^1–3^. In this field, interaction among the light and electrons by considering their spin degree of freedom in the absence of an external bias is studied. In modern optoelectronics, a large number of theoretical and experimental works have been performed by considering optoelectronic and spintronic properties, simultaneously^4–6^. Several approaches have been proposed to produce spin current using optical absorption^7,8^.

Successful synthesis and peculiar optical, electrical and spin transport features of graphene paved the way for utilizing of other two dimensional (2*D*) graphene-like materials in optoelectronics applications. General name for 2*D* buckled monolayer structures which are formed by other group-IV elements is *X*enes (silicene (Si-based), germanene (Ge-based) and stanene (Sn-based)). The stable structure of the buckled *X*enes in comparison with graphene is a consequence of a $sp^{2}{-}sp^{3}$ mixed hybridization of Si, Ge, or Sn atoms in the forming process of these materials^9,10^. The layer separation between two triangular sub-lattices in this group of 2*D* materials is called buckling height^11^. This buckled structure allows us to tune the band gap size by applying a vertical electric field^12–14^. The existence of strong spin-orbit coupling introduce *X*enes as (2*D*) topological insulators (*TIs*) . *TIs* are novel states of quantum matter which have outstanding properties for applications in fundamental condense matter physics and material science^15,16^ . These materials are featured with an insulating band gap in the bulk and conducting state at the edges protected by time-reversal symmetry. Different topological phases are introduced such as the quantum spin Hall (*QSH*) effect, the quantum anomalous Hall (*QAH*) effect and quantum-spin-quantum-anomalous Hall (*SQAH*) effect. Actually, the *QAH* effect, *QSH* effect, and *SQAH* effect can be observed in*X*enes under the effect of external fields. The peculiar feature of topological edge states are proposed to investigate the transport property, which is a platform for spintronic applications that employs the spin degree of freedom^17–20^. One of the outstanding materials in this 2*D* material family is stanene. Recently, ultrathin Sn films with 2*D* stanene structure have been observed on the substrate of $\text{{Bi}}_{2}\text{{Te}}_{3}$ by molecular beam epitaxy experiment^21^. Also, other dominant physics features such as large-gap 2*D* quantum spin Hall states^22^, giant magnetoresistance, perfect spin filter^23^ and near-room-temperature quantum anomalous Hall effect^24^ have been reported for stanene.

Besides spintronic applications, stanene nanostructures are shown to be promising candidates for optoelectronic devices, and there have been important advances in this field during the last decade. Recently, electromagnetic response of staggered 2*D* lattices is studied via an external electrostatic field and circularly polarized laser light. It has been found that different topological phases in these lattices exert influence on the resonant behavior of nonlocal Hall conductivity, considerably^25^. Using density functional calculations, electronic and optical properties of the graphene/stanene heterobilayers are investigated. Combination of the high carrier mobility of graphene and excellent spin Hall effect of stanene in these heterobilayers facilitate the performance of stanene related spintronic devices and suggest this material as a suitable candidate for photoelectronic devices^26^. In a previous study using work function computations, it is revealed that stanene and doped stanene have lower work function in comparison with graphene and accordingly stanene is a good candidate for photocatalysis devices^27^.

Mechanical control or so-called strain engineering is reported as a suitable procedure for tuning optical and electronic properties in 2*D* allotrope of group-IV elements^28–31^. Due to the special structure of stanene, it seems possible that one can tune stanene’s electronic properties by applying external strains. In this regard, some studies have been performed. By taking into account many-body effects, optical properties of stanene and stanane (fully hydrogenated stanene) have been investigated by applying strain. Strain induced optical gap of stanane provides valuable information on the potential application of stanene optoelectronic devices such as solar cells^32^. Furthermore, continuous evolution of the electronic bands of stanene across nanoribbon is reported under the effect of strain field gradient^32^.

Besides the strain, there have been performed numerous studies on the band structures engineering and conductance properties of 2*D* materials. The edge potential is regarded as an efficient approach for engineering of electronic structure. Specially, Rachel and Ezawa observed quantum spin Hall effect without edge states only by employing different perturbations at the edges of silicene nanoribbons^23^.

Inspired by the increasing attention paid to spintronic and optoelectronic, in this study spin-dependent optoelectronic properties of antiferromagnetic zigzag stanene nanoribbons (*ZSNRs*) are investigated theoretically at room temperature. Electric field spin-polarized photocurrent is computed by means of the self-consistent nonequilibrium Green’s function (*NEGF*) formalism and the tight-binding Hamiltonian under the effect of a linearly polarized illumination. In order to improve the performance of the system, effects of some external fields, such as edge potential and uniaxial strain are studied. The results show that applying a vertical electric field at two edges of the antiferromagnetic device results in band bending effect in the corresponding spin-resolved band structure of *ZSNR*. It was shown that with the narrowing of edge potentials, one could broaden the acceptable photoresponsivity in a broad range of incident photon energies as well as increase optical spin polarization percent. Finally, the effect of uniaxial strain on the optical spin transport properties of the antiferromagnetic nanodevice in the presence of edge potential is studied. The numerical results reveal nearly full optical spin-filtering properties under the effect of different strains.

## Methods

The proposed spin optoelectronic device is designed based on *ZSNR* in the presence of some external field, such as normal electric field, antiferromagnetic field and linearly polarized light field. The performed simulations are divided into two self-consistent computations, in which the first part is based on calculating spin-dependent electron features of *ZSNR* and the second part taking into account the quantum transport equation of the interaction of light with matter by employing the *NEGF* approach. It is worthy to mention that due to the absence of impurity or the electron-phonon interaction in the present study, spin-flip mechanisms are neglected^33^. Consequently, transport equations have been solved for spin-up and spin-down components, individually.

The total Hamiltonian of the proposed nanodevice is divided as follows:

1 $$\begin{aligned} H_{T}=H_{L}+H_{R}+H_{C}+H_{CL}+H_{CR}, \end{aligned}$$

where the first two contributions in Eq. (1) are the Hamiltonian of semi-infinite left and right leads, respectively. $H_{LC}$ and $H_{RC}$ describe coupling between the scattering region and the left and right leads. $H_{C}$ represents the Hamiltonian of scattering region:

2 $$\begin{aligned} H_{C}=H_{0}+H_{e\gamma }. \end{aligned}$$

$H_{0}$ is the tight-binding model in the presence of antiferromagnetic field and the normal electric field as follows^34–36^:

3 $$\begin{aligned} H_{0}= & {} -t\sum _{\langle \,i,j \rangle , \alpha }c^{\dagger }_{i\alpha }c_{j\alpha }+i\frac{\lambda _{so}}{3\sqrt{3}}\sum _{\langle \langle \,i,j \rangle , \alpha ,\beta }\nu _{ij}c^{\dagger }_{i\alpha }\sigma ^{z}_{\alpha \beta }c_{j\beta } \nonumber \\{} & {} +e\ell E_{z}\sum _{i,\alpha }\xi _{i}c^{\dagger }_{i\alpha }c_{i\alpha }+M_{AF}\sum _{i,\alpha ,\beta }\xi _{i}c^{\dagger }_{i\alpha }\sigma ^{z}_{\alpha \beta }c_{j\beta }, \end{aligned}$$

where *t* is the usual nearest-neighbor hopping in the scattering region and its value is equal to 1.3 eV. $c_{i\alpha }(c^{\dagger }_{i\alpha })$ annihilates (creates) an electron with spin polarization $\alpha$ at atom *i*, and $<i,j>$ ($<<i,j>>$) represents the sum over nearest (next-nearest) Sn–Sn pairs. The second term in Eq. (3) accounts for the effective spin orbit coupling parameter with a coupling strength of $\lambda _{so}=100\,\text{{meV}}$. $\vec {\sigma }=(\sigma _{x},\sigma _{y}, \sigma _{z})$ is the Pauli’s spin matrix. $\nu _{ij}=- 1(+ 1)$ for clockwise (anticlockwise) next-nearest-neighboring hopping. The third contribution denotes the effect of a perpendicular electric field $E_{z}$. Also, $\ell$ is the buckling height and $\xi _{i}$ is equal to $+ 1(- 1)$ for the upper (lower) sublattice. The last term is related to antiferromagnetic exchange field^37,38^. The antiferromagnetic region can be realized with two ferromagnetic layers oriented antiferromagnetically on two sides of the sample, for example, Crl3^39–42^ or even can be realized in four layer configuration of two ferromagnetic layers^43^. $H_{e\gamma }=\frac{e}{m_{e}}\,\vec {A}\,.\,\vec {P}$ indicates the electron–photon interaction and it is considered as the first-order perturbation Hamiltonian. $m_{e}$ is the mass of electron, $\vec {A}$ and $\vec {P}$ represent the time-dependent electromagnetic vector potential and the momentum of the electron, respectively^44,45^.

After computing the Hamiltonian of the scattering region and the left and right leads, the retarded Green’s function of the nanodevice, in the presence of light radiation, can be written as:

4 $$\begin{aligned}{} & {} G_{\sigma }(E)=\left[ (E+i\eta )I-H_{C,\sigma }-\Sigma _{T,\sigma }\right] ^{-1}, \end{aligned}$$

where

5 $$\begin{aligned}{} & {} \Sigma _{T,\sigma }=\Sigma _{L,\sigma }+\Sigma _{R,\sigma }+\Sigma _{\gamma ,\sigma }. \end{aligned}$$

In Eq. (4), $\eta$ and *I* are infinitesimal broadening and identity matrix, respectively. $\Sigma _{L(R),\sigma }$ is the retarded self-energy due to the presence of the left and right leads. While the self-energies of the left and right contacts were computed by the Sancho iterative approach^46,47^, the electron–photon scattering is included as self-energy term. In Eq. (5), $\Sigma _{\gamma ,\sigma }$ is the self-energy of the electron–photon interaction which is expressed as:

6 $$\begin{aligned} \Sigma _{\gamma ,\sigma }=\frac{-i}{2}\left[ \Sigma _{\gamma ,\sigma }^{<}(E)-\Sigma _{\gamma ,\sigma }^{>}(E)\right] . \end{aligned}$$

$\Sigma _{\gamma ,\sigma }^{<}$ and $\Sigma _{\gamma ,\sigma }^{>}$ are the lesser and greater self-energies of the electron–photon scattering which are given as follows:

7 $$\begin{aligned} \Sigma _{\gamma ,\sigma }^{\gtrless }(E)= & {} (N_{\gamma }+1)M^{\gamma }G_{\sigma }^{\gtrless }(E^{\mp })M^{\gamma } \nonumber \\{} & {} +N_{\gamma }M^{\gamma }G_{\sigma }^{\gtrless }(E^{\pm })M^{\gamma }. \end{aligned}$$

In the above equation, $E^{\pm }=E\pm \hslash \,\omega$ and $N_{\gamma }$ exhibits the number of photon with energy $\hslash \,\omega$^48^. $M^{\gamma }$ is the electron-photon interaction arising from the perturbation Hamiltonian $H_{e\gamma }$. Each element of $M^{\gamma }$ is given by:

8 $$\begin{aligned} M_{lm}^{\gamma }=\langle \,l|\vec {P}.\,A_{0}\,\hat{e_{p}}|\,m\rangle . \end{aligned}$$

$A_{0}$ is the amplitude of electromagnetic vector potential and it’s direction is determined by the light polarization ($\hat{e_{p}}$). $G_{\sigma }^{<}$ is the electron correlation function^49^:

9 $$\begin{aligned} G_{\sigma }^{<}(E)= & {} G_{\sigma }(E)\left[ \Gamma _{L,\sigma }(E)\,f_{L}(E)+\Gamma _{R,\sigma }(E)\,f_{R}(E)\right. \nonumber \\{} & {} \left. +\Sigma _{\gamma ,\sigma }^{<}\right] G_{\sigma }^{\dagger }(E), \end{aligned}$$

and the hole correlation function is:

10 $$\begin{aligned} G_{\sigma }^{>}(E)= & {} G_{\sigma }(E)\left[ \Gamma _{L,\sigma }(E)\,f_{L}(E)+\Gamma _{R,\sigma }(E)\,f_{R}(E)\right. \nonumber \\{} & {} \left. +\Sigma _{\gamma ,\sigma }^{>}\right] G_{\sigma }^{\dagger }(E). \end{aligned}$$

In Eqs. (9) and (10), $f_{L}(_{R})$ is the left (right) Fermi-Dirac function. $\Gamma _{L(R),\sigma }= i(\Sigma _{L(R),\sigma }-\Sigma _{L(R),\sigma }^{\dagger })$, represents the broadening functions of the left (right) electrode. $\Sigma _{\gamma ,\sigma }$ is determined self-consistently by the iteration method. Once convergence is obtained, one can calculate the spin photocurrent across the system by^50^:

11 $$\begin{aligned} I_{\gamma ,\sigma }= & {} \frac{2e}{\hslash }\int \,\frac{dE}{2\pi }\,Tr\left[ G_{(1,1),\sigma }^{>}(E)\Gamma _{L,\sigma }(E)\,f_{L}(E)\right. \nonumber \\{} & {} \left. -G_{(1,1),\sigma }^{<}(E)\Gamma _{L,\sigma }(E)\,(1-f_{L}(E))\right] , \end{aligned}$$

where $G_{(1,1),\sigma }^{>}(G_{(1,1),\sigma }^{<})$ is the first block of the hole (electron) correlation function^51^.

Spin-dependent quantum efficiency can be written as:

12 $$\begin{aligned} \eta _{\sigma }=\frac{E_{ph}\,I_{ph,\sigma }}{e\,A_{D}\,I_{w}}\times 100\%\,(\sigma =up,\,down), \end{aligned}$$

where $A_{D}=L_{ch}\,W_{ch}$ and $E_{ph}$ are cross section of central channel and photon energy, respectively. Also, spin polarization is defined as $SP(\%)=(|I_{ph,\sigma }|-|I_{ph,-\sigma }|)/(|I_{ph,\sigma }|+|I_{ph,-\sigma }|) \times 100\%$

## Results and discussion

### Spin-polarized photocurrent

Spin-photocurrent across the antiferromagnetic single layer zigzag stanene nanoribbons was simulated through the tight-binding approximation and the *NEGF* formalism by considering the combining effect of the normal electric field and the linearly polarized light. The incident light is monochromatic with constant intensity of $I_{w}=100\,\frac{\text{{kW}}}{\text{{cm}}^{2}}$, which is radiated normally on the top of central channel. In this study, the scattering region has constant length which consisting of 120 unit cells which is sandwiched between two semi-infinite left and right leads. In addition, the scattering region, the left and right leads have same structure.

**Figure 1:**
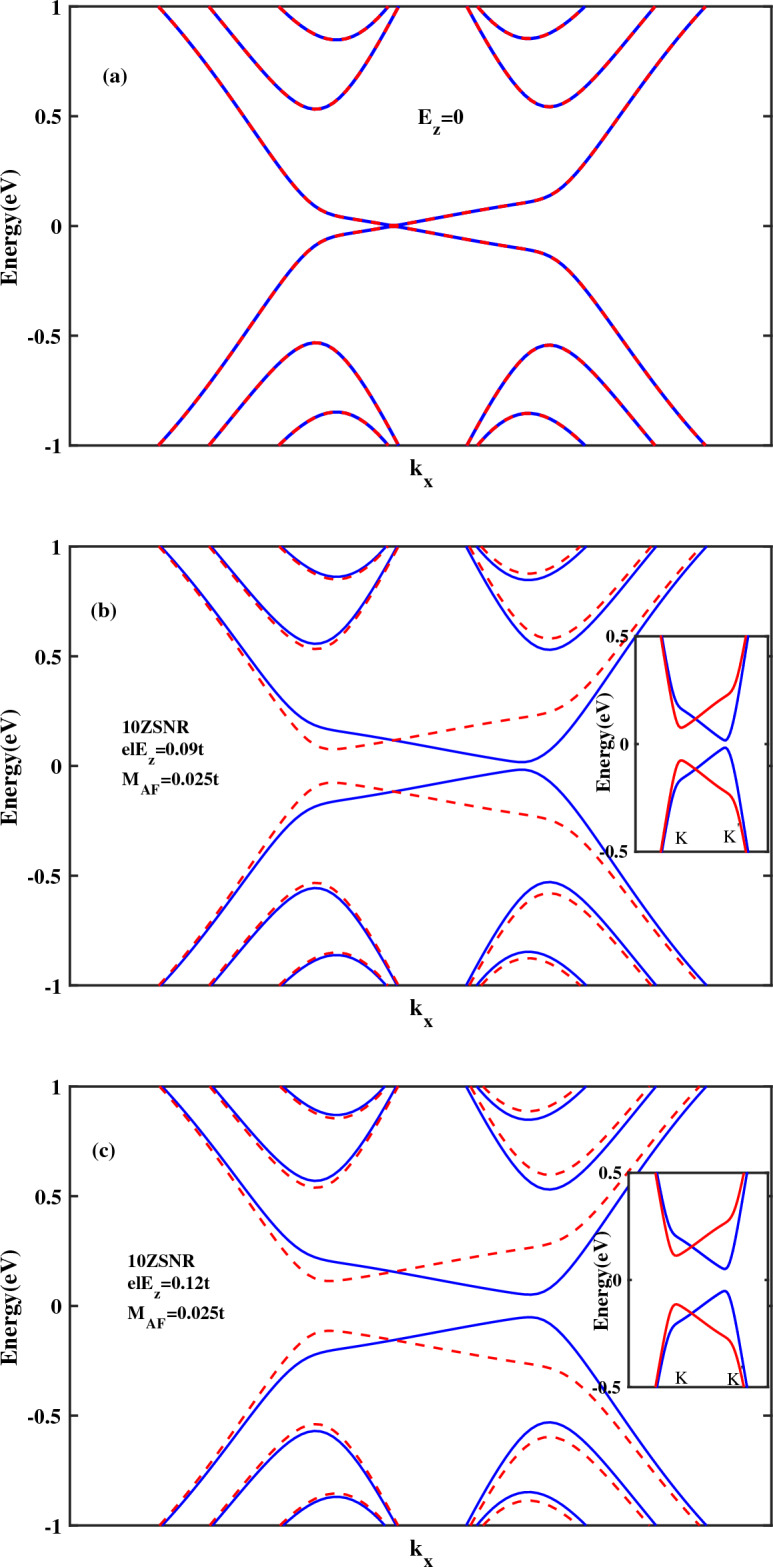
The band structure of antiferromagnetic 10*ZSNR* subject to a perpendicular electric field and $\lambda _{so}=100\,\text{{meV}}$: (**a**) with $E_{z}=0$, (**b**) with $el E_{z}=0.09t$ and (**c**) with $el E_{z}=0.12t$. Blue line denotes spin down and dashed red line denotes spin up.

In the first instance, spin transport properties of *ZSNR* with $N=10$ zigzag chains and 20 atoms in the unit cell are investigated. To this end, spin-resolved band structures of the antiferromagnetic *ZSNR* for various strengths of $E_{z}$ is shown in Fig. 1. These results are similar to reports of previous study^52^. As can be seen in Fig. 1a, in the absence of $E_{z}$, twofold spin degeneracy of the band structure is observed. Applying the external electric field breaks inversion symmetry. The inversion symmetry breaking in combination with the large spin-orbit coupling lifts the band degeneracy in stanene. Also, it is found that switching the direction of the normal electric field will reverse the spin polarization of the band structure and hence, sign of the spin polarized photocurrent. Moreover, finite energy band gap is emerged between spin-up and spin-down energy levels (Fig. 1b,c). As depicted in these figures, the magnitude of band gap is different for spin-up and spin-down energy levels. In the absence of antiferromagnetic term, the magnitude of band gap is equal for both spin states. Physically, due to different effect of the antiferromagnetic term on the potential energies of spin states in the on-site terms of the Hamiltonian, it is expected unequal displacement of spin-up and spin-down energy levels. Accordingly, the presence of the antiferromagnetic exchange field leads to asymmetric spin-dependent band gap and breaking of time-reversal symmetry^38^.

**Figure 2:**
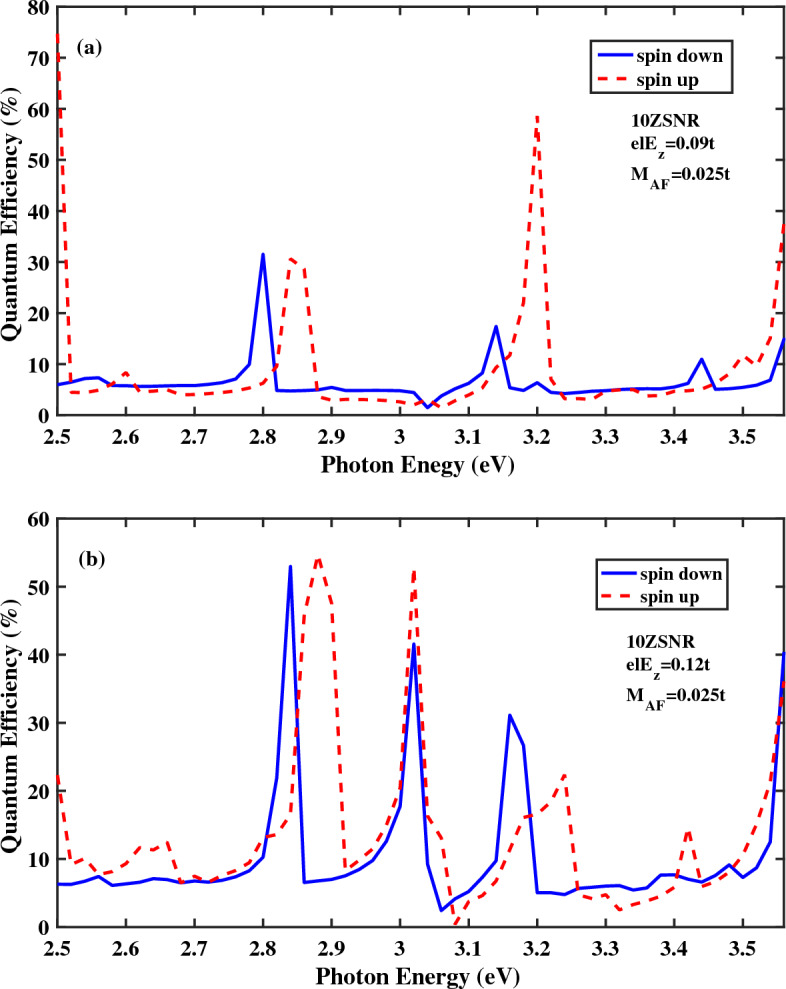
Quantum efficiency as a function of the photon energy for the spin-photovoltaic device based on antiferromagnetic 10*ZSNR* with $M_{AF}=0.025t$ under the simultaneous effect of linear illumination with $I_{w}=100\,\frac{\text{{kW}}}{\text{{cm}}^{2}}$ with $\lambda _{so}=100\,\text{{meV}}$ and (**a**) $el E_{z}=0.09t$, (**b**) $el E_{z}=0.12t$.

The spin-polarized quantum efficiency of 10*ZSNR* with $M_{AF}=0.025t$ versus photon energy under the linearly polarized light and for different strengths of $E_{z}$ is plotted in Fig. 2. As presented explicitly in the inset of Fig. 1b, the asymmetry of band gap energy at the *K* and the $K^{'}$ valleys results in unequal absorption of light which leads to spin population imbalance in these valleys. It can be said that these valleys in *ZSNR* are approximately equivalent to the valleys of infinite stanene sheet which are around the Dirac points^53^. Also, spin splitting of the energy band structure gives rise to spin-dependent absorption when the linearly polarized illumination is shed normally on the top of central region. On the other hand, because of the relatively large enough spin relaxation time in stanene^33^, the electron preserves its spin in this photo-excited phenomena. In fact, the spin-polarized carriers are excited from spin up(down) valence sub-bands to spin up(down) conduction sub-bands by photons with an appropriate energy. Based on these discussions, different occupation numbers between the *K* and the $K^{'}$ ($K^{'}=-K$ ) valleys, which has been induced by the antiferromagnetic exchange field, leads to a unequal spin-polarized photocurrent for spin-up and spin down components. It should be mention that the normal electric field and the antiferromagnetic exchange field is applied to the scattering region.

As can be observed in Fig. 2, the acceptable quantum efficiency is obtained for the photon energy within the range $2.5\,\text{{eV}}< E_{ph} <3.56\,\text{{eV}}$. From the results represented in Fig. 2, one can see considerable variations in spin optoelectronic properties of *ZSNR* in the whole allowable photon energy range. In addition, position and magnitude of the most probable optical transitions are varied by different strengths of $E_{z}$. It is obvious that the electric field changes magnitude of the first optical absorption considerably.

**Figure 3:**
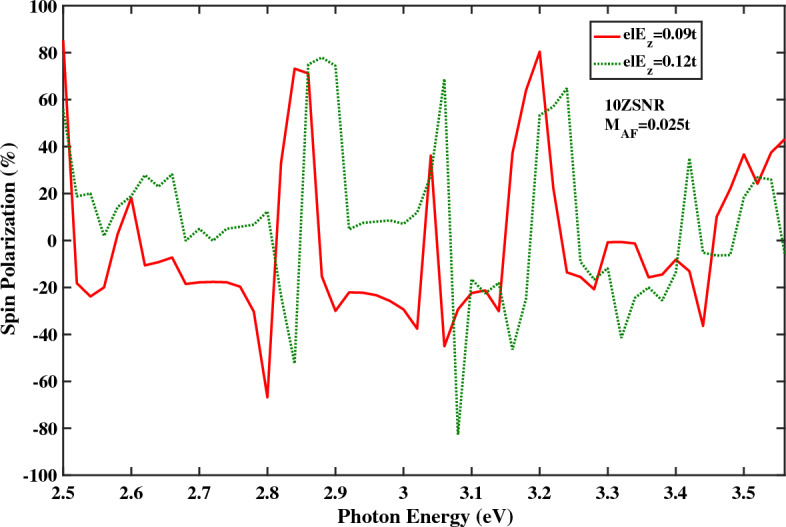
The optical spin polarization as a function of the photon energy for the spin-photovoltaic device based on antiferromagnetic 10*ZSNR* with $M_{AF}=0.025t$ with $\lambda _{so}=100\,\text{{meV}}$ for various $el E_{z}$ strengths.

For more clarification, optical spin polarizations of *ZSNR* for various strengths of $E_{z}$ is displayed in Fig. 3. The spin polarization diagrams for different magnitudes of the electric field have similar qualitative behavior, nearly.

Moreover, spin optoelectronic properties of other narrow ribbons with various widths has been studied. The results reveal similar qualitative behavior but with differences in the magnitudes, positions of the highest peaks of quantum efficiency and acceptable range of the photoresponsivity. These results demonstrate the robustness of the obtained results to the ribbon size. It is worth mentioning that the temperature has been set in the Fermi-Dirac distribution function as room temperature and the other temperature phenomena have been ignored. Of course, it is obvious that the effect of temperature in the form of phonon scattering can modify the obtained results.

### Band bending

**Figure 4:**
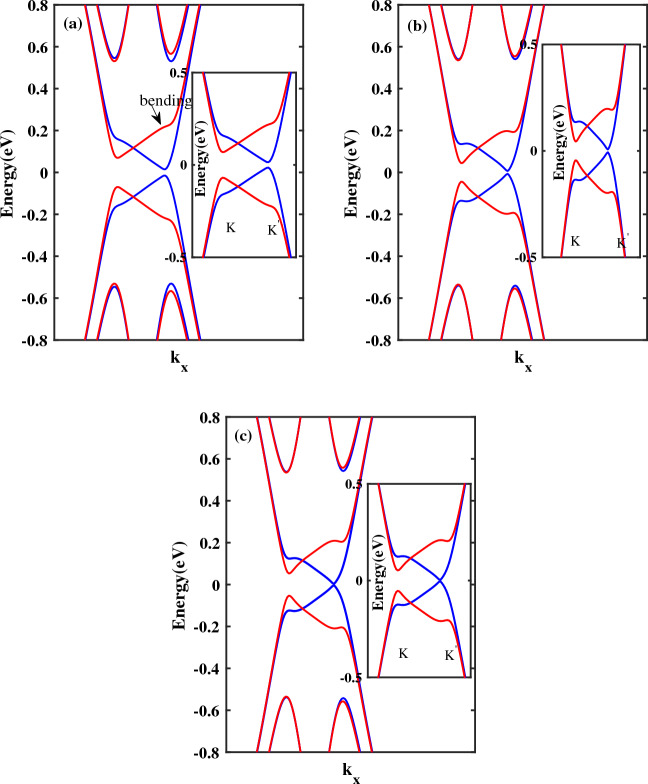
The band structure of antiferromagnetic 10*ZSNR* subjected to a edge electric field and $\lambda _{so}=100\,\text{{meV}}$: (**a**) with $M_{AF}=0.025t$ and applied electric field to $N=8$ zigzag chains, (**b**) $M_{AF}=0.025t$ and applied electric field to $N=4$ zigzag chains, (**c**) $M_{AF}=0.03t$ and applied electric field to $N=4$ zigzag chains. Blue line denotes spin down and red line denotes spin up. Other parameters are: $M_{AF}=0.025t$ and $el E_{z}=0.09t$. Here, $el E_{z}=0.09t$.

In this section the effect of band bending on the spin transport properties of single layer antiferromagnetic *ZSNR* is investigated. A previous study on the band structure and conductance of a zigzag silicene nanoribbon has shown that band bending could be created and controlled by the edge potentials. The bending near valleys can be realized by the edge states^54^. In this paper, we proceed further by studying the benefits of both 2*D* buckled structure and antiferromagnetic spintronics in order to harnessing spin dependent transport in narrow *ZSNRs* in the presence of band bending. To this end, firstly, the band structures of 10*ZSNR* in the presence of edge electric field is analyzed (Fig. 4). In Fig. 4a, the edge field is applied to $N=8$ zigzag chains, such that two chains which are located at the center of ribbon are not affected by $E_{z}$. Also, in Fig. 4b, the edge potential is applied to $N=4$ zigzag chains and six chains at the center of ribbon are not affected by $E_{z}$. It should be noted that applied edge fields at the two edges of ribbon are symmetric, that is, $\vec {E_{z1}}= \vec {E_{z2}}=\vec {E_{z}} \vec {z}$. Owing to the buckled structure of stanene, the edge potentials could significantly impact on the edge states and the band structure. Figure 4a,b indicate that with the narrowing of edge field, the bending enhances gradually, which is in good agreement with previous report^54^. Also, in Fig. 4c electronic band structure of 10*ZSNR* with narrow edge field and $M_{AF}=0.03t$ is displayed. By increasing the magnitude of antiferromagnetic exchange field from $M_{AF}=0.025t$ to $M_{AF}=0.03t$ in combination with applied edge field to $N=4$ chains, the half-metal behavior is revealed at the band structure as shown in Fig. 4c. For spin-down electrons, the gap is closed and metallic behavior is observed, while spin-up carries display still semiconductor properties.

**Figure 5:**
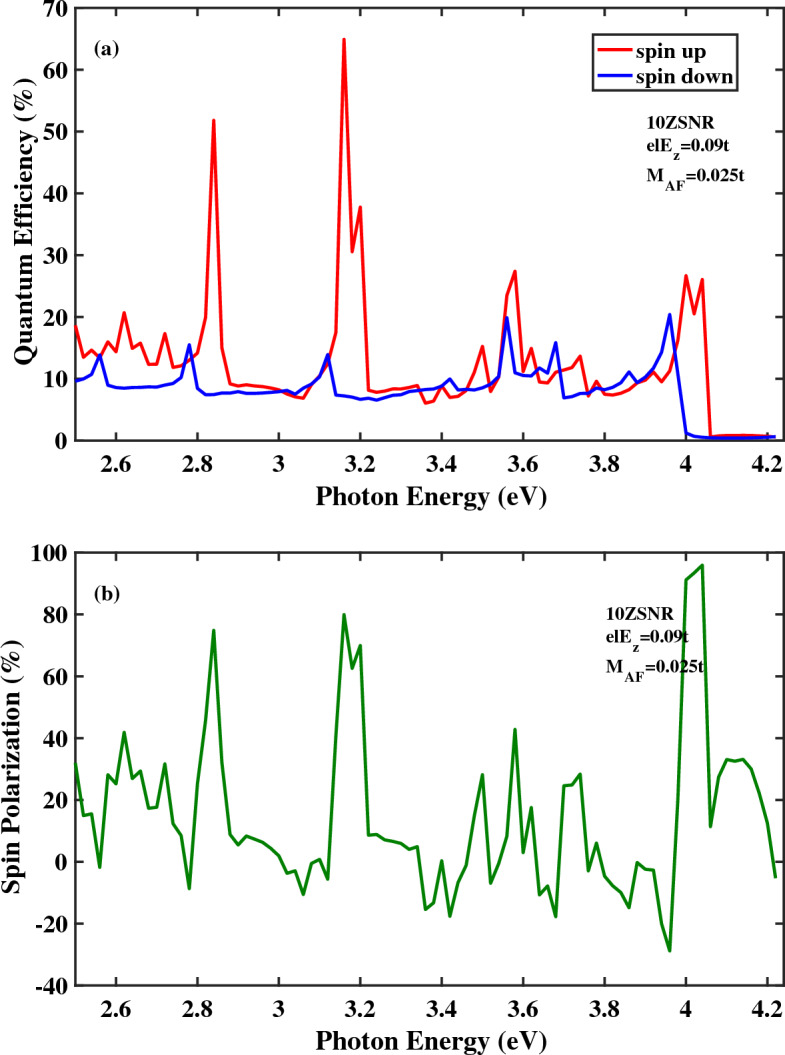
(**a**) Quantum efficiency and (**b**) spin polarization as a function of the photon energy for the spin-photovoltaic device based on antiferromagnetic 10*ZSNR* under the simultaneous effect of linear illumination with $I_{w}=100\,\frac{\text{{kW}}}{\text{{cm}}^{2}}$ and applied edge field to $N=8$ chains. Other parameters are: $M_{AF}=0.025t$, $\lambda _{so}=100\,\text{{meV}}$ and $el E_{z}=0.09t$.

**Figure 6:**
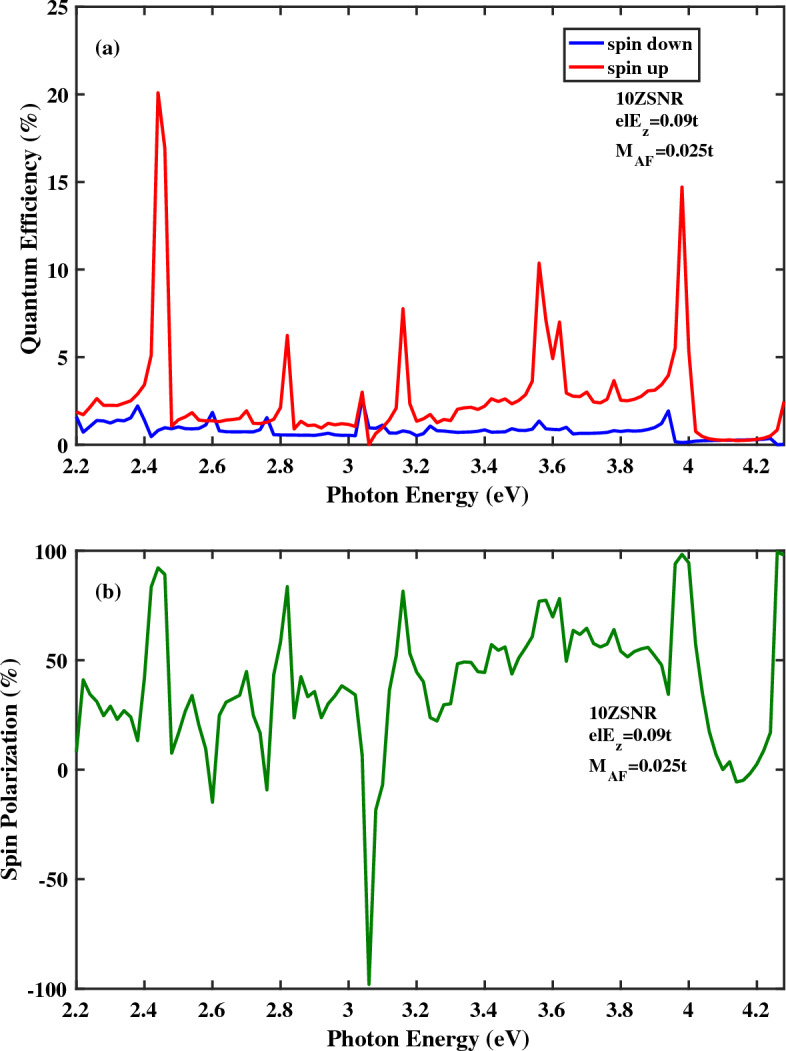
(**a**) Quantum efficiency and (**b**) spin polarization as a function of the photon energy for the spin-photovoltaic device based on antiferromagnetic 10*ZSNR* under the simultaneous effect of linear illumination with $I_{w}=100\,\frac{\text{{kW}}}{\text{{cm}}^{2}}$ and applied edge field to $N=4$ chains. Other parameters are: $M_{AF}=0.025t$, $\lambda _{so}=100\,\text{{meV}}$ and $el E_{z}=0.09t$.

**Figure 7:**
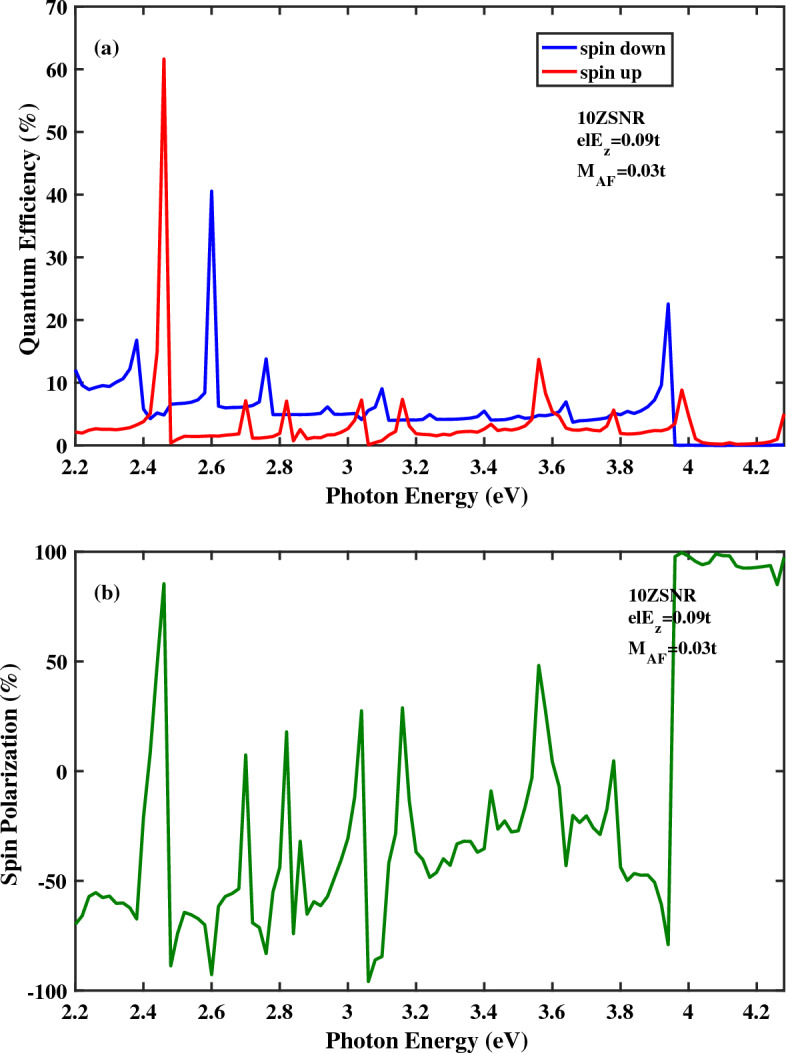
(**a**) Quantum efficiency and (**b**) spin polarization as a function of the photon energy for the spin-photovoltaic device based on antiferromagnetic 10*ZSNR* under the simultaneous effect of linear illumination with $I_{w}=100\,\frac{\text{{kW}}}{\text{{cm}}^{2}}$ and applied edge field to $N=4$ chains. Other parameters are: $M_{AF}=0.03t$, $\lambda _{so}=100\,\text{{meV}}$ and $el E_{z}=0.09t$.

Quantum efficiency as a function of photon energy for the spin-photovoltaic device based on antiferromagnetic 10*ZSNR* under applied edge field to $N=8$ chains is presented in Fig. 5. Note that, here, $el E_{z}=0.09t$ and $M_{AF}=0.025t$. In comparison with Fig. 2a, it is clear that , in general, quantum efficiency is enhanced for both spin states in the presence of edge potential. Note that, in Fig. 2, the electric field is applied to the whole area of the central region. Furthermore, acceptable range of the photoresponsivity is broaden under edge potential. In this case, the maximum magnitude of the spin polarization is obtained $95.94\,\%$. at $E_{ph}=4.04\, {\text{eV}}$. In Fig. 6a,b, quantum efficiency and spin polarization of 10*ZSNR* under applied edge field to $N=4$ chains are computed, respectively. Other parameters are: $el E_{z}=0.09t$ and $M_{AF}=0.025t$. By comparing Fig. 6a with Fig. 5a, it is clear that allowable range of the photoresponsivity is broaden with the narrowing of the edge field. Additionally, the identical qualitative manner of the quantum efficiency is realized, although position and height of the spin-dependent optical transition lines are different, which comes from the difference in the spin-resolved band structure for spin-up and spin-down states. By evaluating of the spin polarization diagrams in Figs. 5b and 6b, one can see that spin polarization is improved with the narrowing of the edge potential. The highest spin polarization peak of 99.43% is appeared at $E_{ph}=4.26\, {\text{eV}}$. The effect of the antiferromagnetic exchange field strength increasing on spin optoelectronic behavior is investigated in Fig. 7. In this figure, the edge potential is applied to the $N=4$ zigzag chains with $el E_{z}=0.09t$ and here $M_{AF}=0.03t$. Obviously in Fig. 7a, quantum efficiency of spin-down carries is higher than quantum efficiency of spin-up carries for a broad range of photon energies which is in contrast to the observed overall trend for quantum efficiency in the presence of the edge field (Figs. 5a, 6a). This distinguished behavior can be attributed to the half-metallic feature of spin-down component. Moreover, as can be observed in Fig. 7b, the perfect $(100\%)$ optical spin polarization is obtained at $E_{ph}=3.98\, \text{{eV}}$.

### Strain

In the crystal structure of a unstrained single-layer stanene and equilibrium condition, each atom of the upper sublattice is connected to its neighboring atoms in the lower sublattice through three bond vectors: $R_{1}^{0}=\frac{a}{\sqrt{3}}(\frac{\sqrt{3}}{2},\frac{1}{2},\cot \varphi )$, $R_{2}^{0}=\frac{a}{\sqrt{3}}(-\frac{\sqrt{3}}{2},\frac{1}{2},\cot \varphi )$ and $R_{3}^{0}=\frac{a}{\sqrt{3}}(0, - 1,\cot \varphi )$, where $a=4.7$ is lattice constant of stanene and $\varphi =107.1^{0}$. The presence of an external tension ${\textbf {T}}$, which is defined as ${\textbf {T}}=T\cos \theta \,\,\hat{e_{x}}+ T\sin \theta \,\, \hat{e_{y}}$, leads to the transformation of these vectors. Longitudinal expansion of stanene layer arising from tension is determined by $\varepsilon =(\acute{a}-a)/a$. The tight-binding approximation is a standard approach to describe the electronic properties of nanodevices. One of the important benefits of this approximation is that one can take into account the effect of strain only by modifying the tight-binding parameters. By applying a uniaxial strain in stanene, equilibrium distance, $R_{0}=a/(\sqrt{3}\sin \varphi )$, is distorted. Thereby, the hopping energy is changed. Calculations demonstrate that under the effect of strain, hopping coefficients are modified as follows^55^:

13 $$\begin{aligned}{} & {} t_{n}(R_{n})=t\left( 1-\beta \,\frac{\delta \,R_{n}}{R_{0}}\right) ,\quad n=1,2,3, \end{aligned}$$

where $\beta$ is Grüneisen parameter and in our simulation we take $\beta =1.95$^56,57^.

To study the effects of a uniaxial strain on the performance of proposed spin-optoelectronic device based on antiferromagnetic ZSNRs, the spin-dependent band structure and the quantum efficiency under various strains is inspected. Experimentally, in 2D materials, local strains are generated by depositing them on a prestretched elastomeric^58^ or rough substrates^59^.

**Figure 8:**
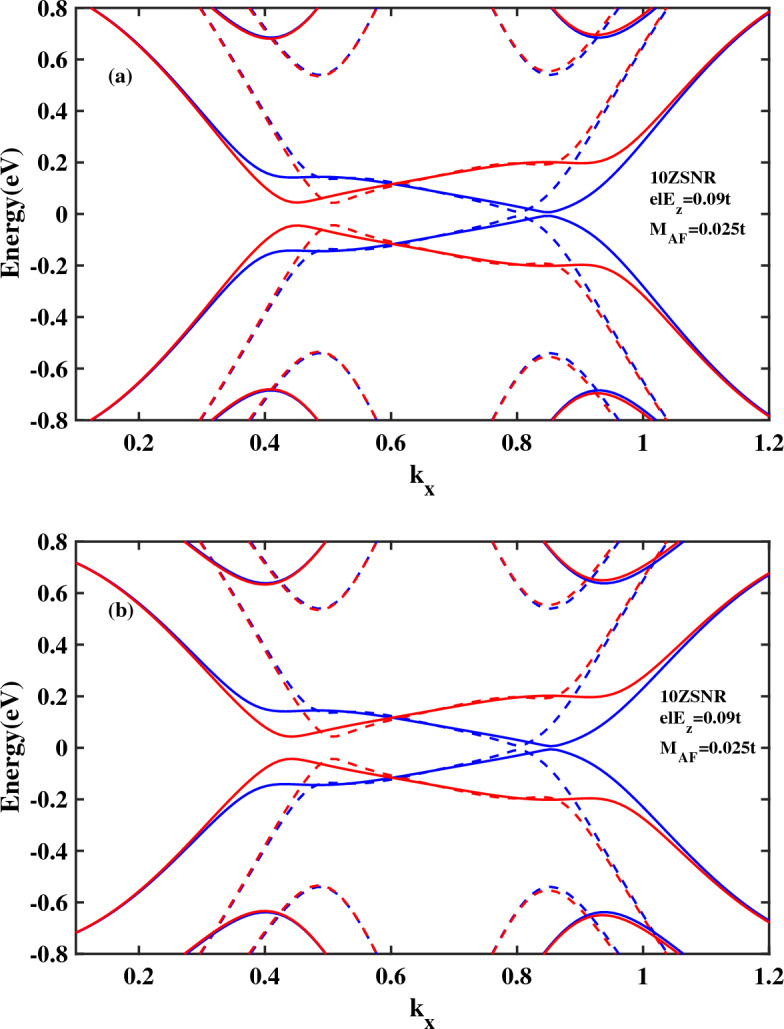
The band structure of antiferromagnetic 10*ZSNR* subjected to the combining effect of strain and narrow edge potential with applied edge field to $N=4$ chains ($\lambda _{so}=100\,\text{{meV}}$). (**a**) $\varepsilon =0.2$ and $\theta =0^{\circ }$ and (**b**) $\varepsilon =-\,0.2$ and $\theta =90^{\circ }$. Blue line denotes spin down and red line denotes spin up. Also, dashed blue line denotes spin down and dashed red line denotes spin up in the absence of strain.

The results mentioned in this sub-section are for the case in which spin-optoelectronic device is based on antiferromagnetic 10*ZSNR* and edge potential is applied to $N=4$ zigzag chains where six chains at the center of ribbon are not affected by $E_{z}$. As depicted in Fig. 8, applying the uniaxial strain leads to a shift in the spin-resolved sub-bands around the Fermi energy and it therefore results in change of population of the states. As a consequence, by employing strain, one can monitor accumulation of spin-polarized carriers in the scattering region. In this case, the variation of the kinetic energy induced by strain in the hopping term of the Hamiltonian is included. This may lead to the alteration of magnitude and also the number of peaks corresponding to spin-up and spin-down components.

**Figure 9:**
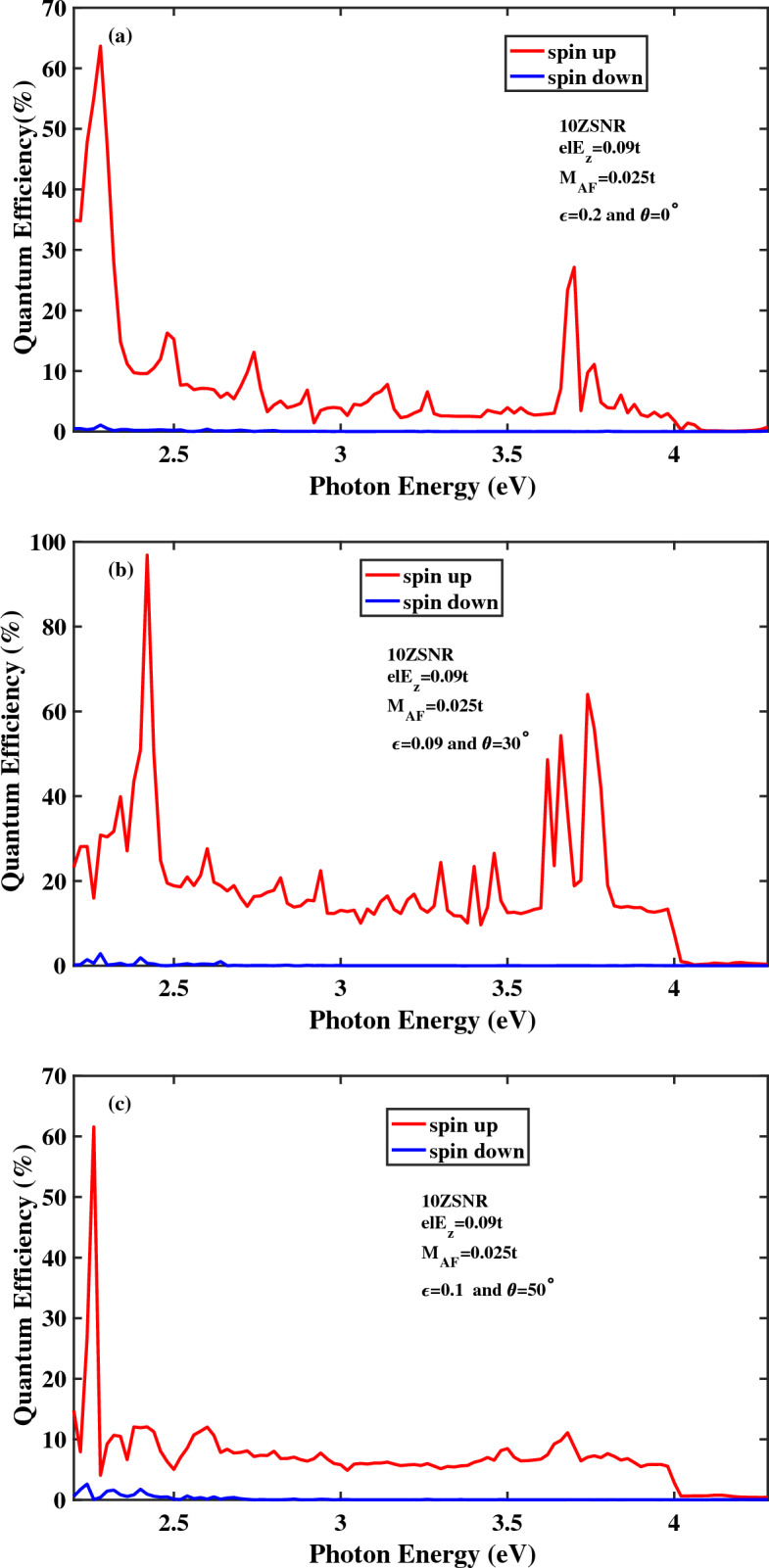
Quantum efficiency as a function of the photon energy for the spin-photovoltaic device based on antiferromagnetic 10*ZSNR* under the combining effect of strain and narrow edge potential with applied edge field to $N=4$ chains. Other parameters are: $I_{w}=100\,\frac{\text{{kW}}}{\text{{cm}}^{2}}$, $M_{AF}=0.025t$, $\lambda _{so}=100\,\text{{meV}}$ and $el E_{z}=0.09t$.

Figure 9 presents the numerical results for the spin-dependent quantum efficiency of 10*ZSNR* for different applied tensions. As can be seen in this figure, different tensions reveal spin-optoelectronic features in an energy range between $2.2\,\text{{eV}}< E_{ph} <4.28\,\text{{eV}}$. In addition, the similar qualitative behavior for the spin-dependent quantum efficiency diagram is obtained in the presence of different strains. Nevertheless, due to the variation in spin-resolved energy levels and the existence of various band gaps for spin-up and spin-down states for different strains, location and height of the spin-dependent optical transition peaks are varied. Moreover, by comparing Fig. 9 with Fig. 6a it can be understood that in the presence of strain the magnitude of quantum efficiency is decreased strongly for spin down component and moderately increased for spin up component. Accordingly, it is expected that applying strain leads to spin-filtering effect in the spin optoelectronic device.

**Figure 10:**
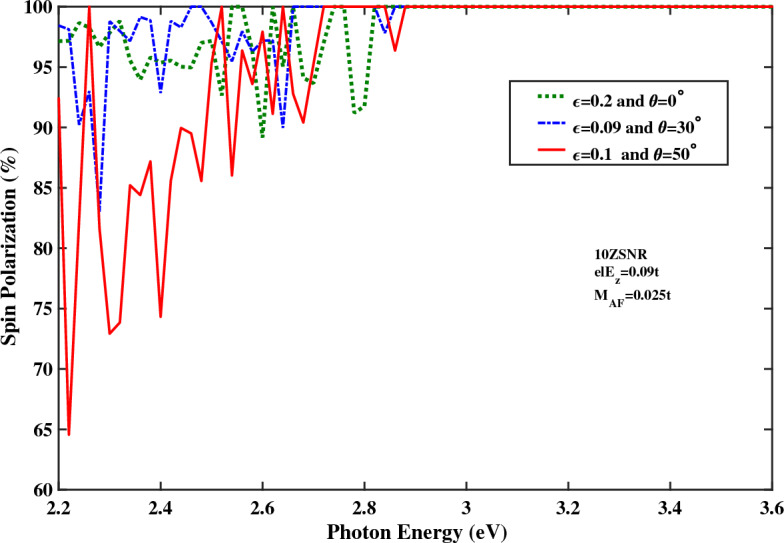
Optical spin polarization as a function of the photon energy for the spin-photovoltaic device based on antiferromagnetic 10*ZSNR* under the combining effect of strain and narrow edge potential with applied edge field to $N=4$ chains. Other parameters are: $I_{w}=100\,\frac{\text{{kW}}}{\text{{cm}}^{2}}$, $M_{AF}=0.025t$, $\lambda _{so}=100\,\text{{meV}}$ and $el E_{z}=0.09t$.

In order to get an exact explanation of spin-filtering effect in the presence of strain, the optical spin polarization of ZSNR as function of the photon energy is shown in Fig. 10 for different strengths of the strain. As mentioned earlier, the spin splitting of the electronic band structures which is attributed to the simultaneous effect of vertical electric field and large spin orbit coupling leads to appearance of the spin polarization and particularly, a fully spin-polarized photocurrent for only one spin state. Furthermore, the spin-filtering photoresponsivity which is induced under the effect of strain is occurred for the wide photon energy range from 2.84 to 4.28 eV, approximately. It can be said that strain improves the optical spin-filtering property of stanene lattice such that the full spin polarization range of the photon energy is broaden considerably.

## Conclusion

In summary, spin-polarized photocurrent in the antiferromagnetic single layer stanene nanoribbon under the perpendicular electric field is theoretically investigated. Optical response of spin optoelectronic nanodevice in the presence of linearly polarized illumination is calculated by means of the self-consistent nonequilibrium Green’s function approach together with the tight-binding model. In antiferromagnetic *SZNR*, twofold spin degeneracy of spin-resolved energy levels is split due to combination effect of the vertical electric field and the large spin-orbit interaction in stanene lattice. Also, robustness of the spin-polarized photocurrent has been demonstrated when the electric field is applied on the two edges. Interestingly, the spin-polarized current is generated in the wide wavelength region of incident light in the presence of the edge potential. Besides, the results indicate that band bending enhances spin-polarization in device. In particular, it is shown that in the presence of the narrow edge potential, by varying antiferromagnetic exchange field, spin-semiconducting behavior is obtained in stanene nanoribbon. This behavior arises as a consequence of the large spin-orbit coupling in stanene lattice. Furthermore, it is found that the spin-resolved photocurrent can be engineered by external strain and nearly full optical spin-filtering is obtained in antiferromagnetic stanene lattice under the combining effect of strain and narrow edge potential. The obtained results in this study may be useful to develop stanene-based nanodevices such as spin-photo detectors, spin photodiodes and generation of full spin-filtering.

## Data Availability

The data and code that support the findings of this study and are used to generate the figures are available upon reasonable request from the corresponding author.
